# Critical Care Management of Severe COVID-19 in Pregnant Patients

**DOI:** 10.7759/cureus.24885

**Published:** 2022-05-10

**Authors:** Hashsaam Ghafoor, Aijaz Abdus samad, Ali O. Mohamed Bel Khair, Osman Ahmed, Muhammad Nasir Ayub Khan

**Affiliations:** 1 Department of Anaesthesiology, Hamad Medical Corporation, Al Khor, QAT; 2 Department of Anaesthesiology, Latifa Women and Children Hospital, Dubai, ARE; 3 Department of Anaesthesiology and Critical Care, Shifa International Hospital Islamabad, Islamabad, PAK

**Keywords:** delivery, critical care, management, pregnancy, covid-19

## Abstract

Since December 2019, the coronavirus disease (COVID-19) pandemic has had a disastrous impact worldwide. COVID-19 is caused by the SARS-CoV-2 virus and was declared a pandemic by the WHO on March 11, 2020. The virus has been linked to a wide range of respiratory illnesses, ranging from mild symptoms to acute pneumonia and severe respiratory distress syndrome. Pregnant women are more vulnerable to COVID-19 complications owing to the physiological and immunological changes caused by pregnancy. According to the CDC, pregnant patients with COVID-19 are commonly hospitalized and often require admission to ICUs and ventilator support. Therefore, it is especially important for pregnant women to adhere to disease prevention measures to lower the risk of contracting the disease. In addition, the guidelines of several clinical societies and local health authorities should be followed when caring for pregnant women with suspected or confirmed COVID-19. In this review article, we discuss the epidemiology of COVID-19 during delivery, its effect on the physiological and immunological changes during pregnancy, the classification of COVID-19 severity, maternal and fetal risks, antenatal care, respiratory management, treatment/medication safety, timing and mode of delivery, anesthetic considerations, and the outcome of critically ill pregnant patients with COVID-19, as well as their post-delivery care and weaning from mechanical ventilation.

## Introduction and background

Coronavirus disease (COVID-19) was first reported in the Chinese city of Wuhan in December 2019 and quickly spread until reaching pandemic status in March 2021 [1]. COVID-19 is caused by SARS-CoV-2 [2], a novel, enveloped RNA coronavirus that can be transmitted between people via contact and droplets [3, 4]. With the spread of coronavirus, pregnant women and their fetuses have been identified as a high-risk population [5]. This review article discusses the critical care management of severe COVID-19 in pregnant patients.

## Review

Epidemiology of COVID-19 during pregnancy

Approximately 29 million individuals worldwide have been infected with COVID-19, with >900,000 deaths (3.2% case fatality rate) [6]. In addition, case-fatality rates of almost 50% have been reported [7].

Pregnant women are at an elevated risk for morbidity and mortality during prior epidemic respiratory illnesses, with data from seasonal influenza, the 2009 H1N1 pandemic, and the SARS-CoV-2 epidemic showing elevated rates of admission to the ICU, intubation, and death compared to non-pregnant patients [8].

The WHO-China Joint Mission for novel COVID-19 reported that among 147 infected pregnant women, including 64 confirmed, 82 suspected, and one asymptomatic case, 8% had severe COVID-19, and 1% had critical disease [9].

In the United States, 39,857 COVID-19 cases were reported among pregnant women, and 8284 (21.0%) required hospitalization/ICU admissions. New York City was the first state to be overwhelmed by the COVID-19 pandemic. Between March 22 and May 01, 2020, among 2256 pregnant hospitalized females, 271 (12.1%) were found to be SARS-CoV-2 positive; among them, 3.1% were symptomatic, while 0.7% required ICU admission [10].

Regarding the effects of COVID-19 during pregnancy, a study conducted among 119 pregnant females and a systematic review among 109 pregnant women demonstrated acute infection rates requiring ICU admission of 2.7%-6.9%, with no mortality cases [11].

Physiological and immunological changes during pregnancy

Physiological changes in the respiratory and cardiovascular systems during pregnancy make women susceptible to pathogen infectivity and acute pneumonia [12]. Pregnant women have an elevated diaphragm, as well as increased heart rate, oxygen consumption, diminished lung volume, increased blood volume, and an edematous respiratory tract mucosa. Vasodilation and changes in lung volume cause high respiratory secretions and airway edema. Hence, pregnant women have a weak tolerance to hypoxia [13]. Owing to suppressive T cell effects, pregnant women have transitory immunosuppression; thus, they are considerably more vulnerable to viral infections [14]. In addition, alterations in cell-regulated immunity make pregnant women more susceptible to intracellular pathogens and viruses [15]. Moreover, Th (T helper) 17 resistance is considerably enhanced among patients infected with COVID-19, resulting in the release of several inflammatory cytokines [16]. Fetuses born from females infected with COVID-19 may be in an inflammatory state, promoted by the placental sample/systemic resistant response of their mothers. The Treg/Th17 resistance balance is most significant during normal gestation [17]. If an increase in Th17 cells and a decrease in Treg cells are observed, it could lead to frequent pregnancy loss, preterm delivery, and preeclampsia [18]. COVID-19 infection-resistant inflammatory reactions can cause several obstetrical complications, resulting in short- and long-term adverse maternal and fetal outcomes [12].

Classification of COVID-19 severity

Asymptomatic

A COVID-19 positive test result without indications [19].

Mild

Flu-like indications, including cough, fever, myalgias, shortness of breath, anosmia with no dyspnea, and atypical chest imaging [19].

Moderate

Confirmation of lower respiratory tract disease by clinical evaluation (pneumonia and dyspnea on imaging, abnormal blood gas findings, refractory fever >39.0 °C, not relieved with paracetamol while maintaining an oxygen saturation >93% in room air at sea level) [19].

Severe

Respiratory rate >30 breaths per minute, hypoxia together with <93% oxygen saturation, a ratio of oxygen in arterial partial pressure to inspired oxygen fraction of <300%, or >50% lung involvement on imaging [19].

Critical

Multi-organ dysfunction/failure, respiratory failure, or shock requiring mechanical ventilation (MV) or high-flow nasal cannula [19].

Approaches in pregnant patients with COVID-19

Screening of Signs and Symptoms

Pregnant women and their attendants’ screening is most important and should be performed at all attendance steps, outpatient pre-and post-partum care, high-risk pregnancy hospitalization, labor, and puerperium [20,21]. In addition, entries must be limited to the maternity hospital to ensure that everyone is screened for COVID-19 [22].

It is essential to create a screening checklist that allows the identification of suspected cases, confirmed cases, or not. When a potentially at-risk patient is admitted, a surgical mask should be worn, and follow-up should be performed preferentially in the hospital’s specific sector. In addition, health professionals should wear adequate personal protective equipment (PPE) [23].

Type of Consultation

Consultations through phone/video conferences can reduce infections in patients, attendants, and healthcare providers. A reduction in the patients’ stay in the health facility environment can be endorsed with guided consultation and essential examination. The use of masks by patients, frequent hand hygiene, and/or the use of 70% alcohol should be stipulated [20].

In patients with negative screening results, in-person consultations can be executed with proper distancing procedures and by providing an extended time. It is essential to maintain in-person consultations because activities such as measuring uterine length, weight, and blood pressure cannot be performed remotely, and reliable devices are not always available at home [20].

Medical Care Location

Suspected and probable COVID-19 cases during pregnancy should be screened. Suspect, probable, and confirmed cases, considered mild and without risk factors, can be handled in primary healthcare, while moderate/severe cases should be forwarded precociously to the tertiary hospitals. Isolation rooms with negative pressures are recommended [20].

Preferentially, maternity hospitals or natural childbirth centers should be selected for the exclusive treatment of suspected, probable, and confirmed cases. In addition, these locations should build specific environments for vaginal delivery and surgery, neonatal isolation in ICUs or intermediate care, and shared accommodation [21].

Attendance Room and Isolation

In suspected, probable, or confirmed cases that require in-person, outpatient/hospital attendance, it is recommended that they be placed in isolation, with a limited circulation of patients and healthcare providers [20]. If the maternity screening sector is contacted before the arrival of pregnant women, the managers of the sector should organize and prepare resources. Ideally, in patients with confirmed COVID-19, a negative-pressure room is necessary; if unavailable, an exclusive room should be used, in which all unnecessary equipment is withdrawn [20].

Disinfection of the room and equipment should be performed according to local protocols [23]. Equipment should be exclusive, such as electronic fetal monitors or ultrasound devices, should not be displaced, and disinfection between patients must be performed [20].

Maternal and fetal risks

A significant impact on the pregnancy of COVID-19 is required, as per the stage/trimester of infectivity, to determine the maternofetal outcome [24].

First Trimester

During the first trimester, a few reported cases attempted to demonstrate an association between abortion and infection; however, this could be accidental, with a 9% abortion incidence among females with COVID-19 versus 11% of the general population [25].

For a likely association, a study was conducted in Barcelona, in which 1,908 and 317 pregnant women with and without SARS-CoV-2 were examined, respectively. Insignificant differences were found in obstetric complications among those diagnosed during the first half of pregnancy with (1.4%) and without (1.9%) infection during the second half of pregnancy. The results further suggested no relationship between abortion and infection during the first half of pregnancy [26].

Second and Third Trimesters

Lam CM et al. proposed that pregnant women with COVID-19 had an elevated rate of intubation, ICU admission, and mortality compared to non-pregnant women with COVID-19; however, no virus transmission was found among infants [27]. Additional complications, such as preterm birth, small gestational age (GA), and miscarriage, have also been described. However, another study showed no increase in the risk of impetuous preterm delivery and abortion among pregnant females with COVID-19 [28].

Several studies have reported infections and inflammatory conditions in relation to preterm delivery among COVID-19 infected females. However, these studies have mainly been from China, which has a dissimilar medical system. It is unclear whether SARS-CoV-2 led to these premature deliveries or if this was iatrogenic because of fetal or maternal distress or some other factors indications [29].

Antenatal care

Maternal Care

All pregnant women should physically distance themselves and follow the guidance regarding self-isolation and good hand hygiene practices to avoid COVID-19 exposure. During a pandemic, general guidelines must be followed to reduce in-person hospital visits, and, if appropriate and practical, appointments should be made through telephone/videoconferencing. Symptoms among women with COVID-19 should be confirmed, and appointments, if possible, should be delayed during the self-quarantine period. If disease symptoms persist, patients should make an appointment regarding testing or hospital admission. Maternity units must consider additional measures, such as limiting the number of visits by patients’ attendants in outpatient/inpatient, labor, and delivery areas [30].

Fetal Surveillance

For women who have been cured of COVID-19, close monitoring by consistent sonographic evaluation is necessary to evaluate the growth and well-being of the fetus [31].

Therapeutic management of COVID-19 in the setting of pregnancy

Oxygen Therapy

During pregnancy, oxygen therapy is a common technique that includes a high-flow nasal cannula and non-rebreather masks [32]. Although pregnant women may suffer considerable nasal blockage, a high-flow nasal cannula still appears to be useful [33]. Limited data are available to identify proper oxygen goals, and numerous references recommend an elevated oxygen level among the non-pregnant population. Although insufficient evidence is available to support this, hyperoxygenation may have unfavorable maternal hemodynamic effects during pregnancy [34].

Non-Invasive Respiratory Support

Non-invasive respiratory support includes helmet and face-mask continuous positive airway pressure and non-invasive divalent ventilation and is extensively utilized for COVID-19 among the non-pregnant population in some areas. This is believed to be a viable strategy, with the potential to avoid intubation [35], and is believed to be safe if the patient is attentive and protects the airway. Aspiration risk should always be considered, and there is a potential advantage in avoiding sedation and intubation. Non-invasive respiratory support has been effectively utilized among pregnant females with COVID-19 [36].

Intubation and Ventilation

It is well recognized that airway management can be challenging during pregnancy, and careful airway evaluation should be performed by a skilled operator, preferably an obstetric anesthesiologist. During pregnancy, concerns associated with airway management include an edematous, friable airway, elevated aspiration risk, aortocaval compression, and hemodynamic volatility requiring displacement of the left uterus [37]. An enhanced risk of swift oxygen desaturation is caused by decreased functional residual volume and increased oxygen intake, which could worsen in patients with COVID-19 hypoxia. No recommended alterations are available regarding rapid sequence induction dosage; however, care should be taken to ensure an appropriate weight-based neuromuscular blocker dosage to facilitate rapid intubation. Video laryngoscopy is recommended for pregnant women with COVID-19 [38].

The principles of MV are the same as those in the non-pregnant population, with a targeted tidal volume of 6 ml/kg based on ideal body weight. No evidence suggests an alteration in the mode of ventilation/alteration of the monitoring parameters when required. However, pregnant women may need an elevated level of positive end-expiratory pressure (PEEP) to obtain alveolar recruitment. The plateau pressure could be slightly higher because of decreased compliance of the respiratory system [39].

**Figure 1:**
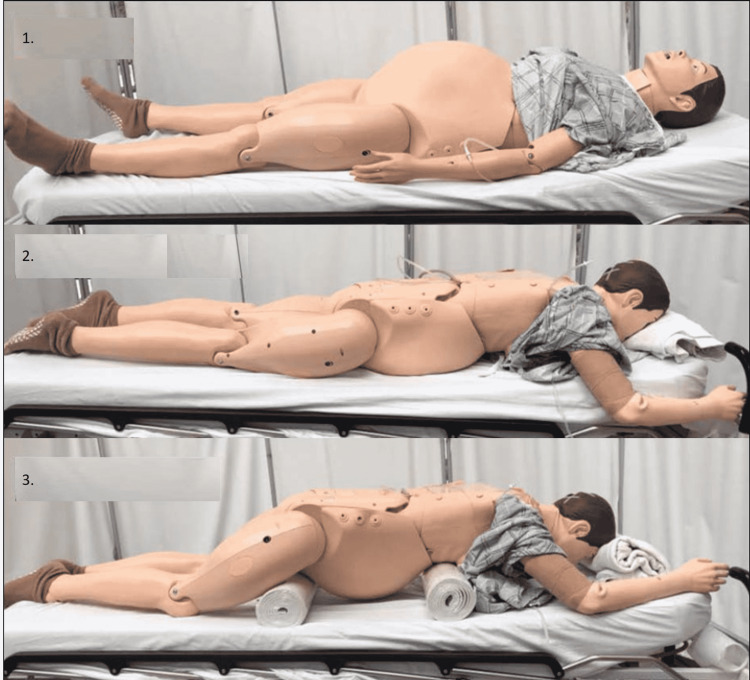
Prone positioning of pregnant patient with key area of support. 1) Supine 2) Prone without support 3) Prone with support With permission from Oxford-Horrey C et al. (2020) [41].

During pregnancy, the supine positioning is effective and feasible [40], while prone positioning must be performed along with chest and hip support to reduce abdominal pressure. Figure 1 shows the key areas in which it is necessary to avoid direct pressure on the pregnant uterus [41]. Inhaled bronchodilators, namely inhaled nitric oxide, can be utilized during gravity. Extracorporeal life support during pregnancy is considered an alternative, with good fetal and maternal outcomes [42].

Blood Gas Targets

Attaining an elevated oxygen target is mainly restricted to maternal pathophysiology, and oxygen saturation is an element of oxygen supply to the fetus. Aiming for oxygen saturation >95% during gravidity is not empirical. Interventions to boost maternal oxygen saturation could decrease cardiac output and placental perfusion, resulting in unfavorable fetal outcomes. The fetus can mitigate hypoxemic effects (even a <50% decrease in oxygen content) by forwarding cardiac output to the brain and heart. An additional drop in oxygen content generates anaerobic metabolism, and CNS damage can occur if the oxygen supply decreases by >75% [32].

Permissive hypercapnia is a recognized technique used to ventilate patients with adult respiratory distress syndrome (ARDS) to reduce injurious TVs (tidal volume) [43]. However, concerns exist among pregnant patients because normal PaCO2 levels are reduced. Some clinical studies have highlighted the lack of unfavorable fetal outcomes from insignificant hypercapnia (40-55 mmHg) [44], while a case series verified successful pregnancy outcomes after short-term significant hypercapnia (PaCO2 43-114 mmHg; 5.7 to 15.2 kPa) [45]. The management of hypercapnia is an important risk-benefit balance between harmful elevated TVs and possible unfavorable outcomes of hypercapnia. PaCO2 is mainly allowed to rise to 50 mmHg (6.7kPa) among such patients. With low levels of PaCO2, hyperventilation decreases uterine blood flow and compromises fetal oxygenation through alkalosis-induced uterine vasoconstriction and decreased cardiac output caused by high intrathoracic pressure (Table 1) [46].

**Table 1:** Mechanical ventilation parameters and recommended management strategies among pregnant females with adult respiratory distress syndrome. ARDS: Adult respiratory distress syndrome; FiO2: A fraction of inspired oxygen; MV: Minute ventilation; P_plat_: Plat plateau pressure; PEEP: Positive end-expiratory pressure; RR: Respiratory rate; TV: Tidal volume. Source: reference [41].

Parameter	Description	Target in ARDS
TV	The volume delivered by a ventilator with each breath	4-8 mL/kg predicted body weight (based upon the patient’s height)
RR	Number of breaths per minute delivered by the ventilator	Minimal RR required to match the baseline MV, which is elevated among pregnant females typically by 30%-40% and driven mostly through enhanced TV during pregnancy MV = TV x RR
P_plat_	Pressure applied to small airways and alveoli measured by an inspiratory pause at end-expiration on the ventilator	P_plat _< 35 cm H_2_O during pregnancy (accounts for pressure from the gravid uterus while reducing volutrauma)
PEEP	Pressure applied to mitigate end-expiratory alveolar collapse	PEEP is applied in combination with FiO_2_ to achieve the desired oxygenation of PaO_2_ 60-80 mmHg or SpO_2 _> 95%
FiO_2_	Fraction of oxygen delivered by the ventilator (room air is 21%)	
PCO_2_	Measured carbon dioxide in arterial or venous blood; Marker of alveolar ventilation; Hypercapnia is a trade-off in low TV lung protective ventilation	The permissive hypercapnia threshold during pregnancy is poorly identified; though, ranges of 50-60 mmHg may be safe

Electronic monitoring of the fetal heart rate (at the correct GA) could help to assess the effects of peculiar blood gases on the fetus [32].

Treatment/Medication safety in pregnancy

Available drug therapies for COVID-19 have not yet been examined in pregnant women; thus, suggestions are made based on the best available but limited data [32].

*Dexamethasone* 

A study on dexamethasone (6 mg/day) during COVID-19 indicated decreased mortality among pregnant women requiring oxygen/MV [47]. For severe COVID-19 pneumonitis, steroid therapy is considered suitable for pregnant women. Dexamethasone traverses the placenta, and for COVID-19 pneumonitis treatment, a medicine that does not affect the fetus is required. For instance, methylprednisolone 32-40 mg daily (equal to prednisolone 40-50 mg daily) is given to complete a steroid course of 10 days [48]. Depending on the GA, steroids (betamethasone or dexamethasone) could initially be utilized to promote fetal lung development; however, such a decision is best directed by the obstetric team [32].

Remdesivir 

In a study that excluded pregnant subjects, remdesivir demonstrated a shorter recovery time among hospitalized patients requiring oxygen therapy [49]. However, there is currently no consensus on its use in treatment guidelines, either among pregnant or non-pregnant women [50]. In a study of 67 women who received remdesivir during pregnancy, no neonatal mortality or congenital abnormalities were observed [51]. Therefore, treatment guidelines differ from discouraging remdesivir usage during gravidity to following the same technique for the non-pregnant population; that is, to be used for patients with moderate COVID-19, but not for those with acute disease or MV [32].

Tocilizumab

A study of tocilizumab, an IL-6 inhibitor, highlighted improved survival among hospitalized patients with COVID-19 together with hypoxia and systemic inflammation [52]. There are some data available regarding pregnancy from the use of tocilizumab during rheumatological conditions, suggesting that it is harmless during pregnancy [53]. However, there is a theoretical risk for neonatal immunodeficiency and a rise in preterm births, despite no reports on fetal deformities [54]. Therefore, most guidelines endorse the use of tocilizumab based on rheumatological safety facts in acute COVID-19 [32].

Monoclonal Antibodies

Monoclonal antibodies target spike proteins and possess scientific benefits in healing COVID-19. Several monoclonal antibodies (casirivimab plusimdevimab, sotrovimab, and bamlanivimab + etesevimab) have been approved for use (or with specific restrictions) in several jurisdictions. Data regarding pregnancy are currently unavailable, but as immunoglobulin G antibodies, these medicines are anticipated to traverse the placenta after neonatal Fc receptor expression from almost mid-gestation. Several guidelines recommend the use of monoclonal antibodies during pregnancy [32].

Thromboprophylaxis

Both pregnancy and SARS-CoV-2 infection are prothrombotic conditions, in which the risk of coagulopathy and thromboembolism is increased [55]. Therefore, Thromboprophylaxis is highly recommended among pregnant women following standard practices with low-molecular-weight heparin [56].

Glycemic Control 

Preexisting and gestational diabetes are considered risk factors for increased severity of COVID-19 during pregnancy and are linked with unfavorable outcomes. In addition, pregnancy transforms maternal glucose homeostasis. This physiological change, combined with an acute illness stress response and the use of corticosteroid therapy, causes an increased risk of hyperglycemia among hospitalized patients with COVID-19. Therefore, glucose levels should be monitored carefully and treated with insulin when required [32].

Vasopressor Therapy

Vasopressors and inotropes probably decrease placental perfusion but could be essential for end-organ perfusion maintenance (including the placenta). If support for vasopressors is needed, this treatment must not be suspended because of concerns about potentially unfavorable outcomes for the fetus [32].

Timing of delivery: When to deliver

When pregnant women infected with SARS-CoV-2 are more than 34 weeks pregnant, delivery timing is easy to manage. Indeed, in the acute type of disease, the birth must occur quickly to provide care to the mother. However, difficulty in decision-making emerges when pregnancy termination can cause premature delivery. The delivery decision cannot simply follow the rules but needs to be discussed on a case-by-case basis with a multidisciplinary team, weighing up the risks and benefits. The team must involve obstetricians, neonatologists, anesthetists, and patients and their spouses. Fetal extraction indications must not appear too early or too late. It has been suggested that a low dose of catecholamine and intubation itself is not a sign of fetal abstraction alone; however, when it takes place, it must lead to close and constant communication among all members of the multidisciplinary team to offer timely delivery if the situation deteriorates [57].

Mode of delivery: How to deliver

Delivery should be conducted in an isolation ward under negative pressure. Nurses and health professionals must be completely safeguarded during the complete delivery process and equipped with goggles, protective clothing, and face masks (N95). General/Intraspinal anesthesia and endotracheal intubation can be adopted for delivery. Epidural/General anesthesia can be adopted for cesarean section (CS) among patients with COVID-19. Before anesthesia induction, constant high-flow oxygen must be administered with a face mask, and fast induction of anesthesia must be adopted to prevent choking cough. Once the patient becomes unconscious, a saline-moist gauze with a double layer should be inserted in the mouth and nose to initiate low TV higher-frequency ventilation to prevent virus dispersion and lung injury due to enhanced airway pressure. During the operation, physicians should avoid patients’ secretions, blood, excretions, aerosols, and amniotic fluid while utilizing surgical instruments. All articles must be sanitized after surgery, and the specimens should be sent for pathological examination. Health personnel who collect specimens should undergo training in this regard [58].

Anesthetic consideration: Regional vs. general

Neuraxial anesthesia is the preferred technique for surgical delivery. Single-shot spinal anesthesia with fentanyl/morphine and bupivacaine is recommended as a one-time approach that ensures protracted postoperative analgesia for the parturient (nausea, vomiting, urinary retention, and itching being tradeoffs for such a combination). However, the most frequently used techniques in hospitals must be practiced. No contraindication was observed regarding the use of the transverse abdominis plane or some other blocks to relieve postoperative pain. If an indwelling labor epidural catheter is working, it may be dosed to provide surgical anesthesia [59].

In cases where general anesthesia is needed, the parturient should be intubated by a skilled anesthesiologist. In addition, utilization of PPE and associated problems, such as misting of goggles, can further enhance problems during the intubation of a parturient [59].

Outcome of critically ill pregnant patients

Maternal Outcome

Maternal mortality: Previous studies have suggested a very low maternal mortality rate among those with identified SARS-CoV-2 compared with those with other SARS and MERS infections [30]. A study of 41 pregnant females with COVID-19 highlighted that the maternal mortality was 0% compared to the 25.8% with SARS-CoV-2 and 28.6% with MERS; however, a later study of 108 pregnancies found no maternal mortality [60,61]. The findings of an Iranian study showed maternal mortality in seven of nine pregnant women with severe COVID-19 [62]. C-section rates: Among females with confirmed SARS-CoV-2, C-section rates have been reported to range from 42.9% [63] to 91-92% in some studies [60,61]. A study conducted by Di Mascio D et al. reported that the C-section rate was >90% among women admitted to hospitals with COVID-19 pneumonia [60]. In contrast, such elevated rates of C-sections do not link females with the mild-moderate disease. It seems that several C-sections were conducted in the interest of the mothers due to maternal respiratory function. Moreover, a recent Italian study reported C-sections among 42 women, while eight C-sections were performed without any indication of COVID-19 infectivity [64].

Fetal Outcome

Preterm delivery: Early reviews showed preterm birth elevated rates, ranging from 41% to 47% [60,61,65]. A systematic review of 33 studies subsequently described a preterm delivery rate of 15.2% in 385 pregnant females with COVID-19 with a GA at delivery ranging from 30 to 41 weeks [66]. Although several preterm births were iatrogenic and for maternal causes, some studies report fetal distress as an indication in several cases [66], while in others, the sign of delivery is uncertain [65]. No sufficient data are available to assess any association between SARS-CoV-2 infectivity in pregnancy and spontaneous preterm labor [60,67].

Vertical transmission: Currently, little evidence is available regarding the vertical transmission of COVID-19 to infants. In an initial study conducted by Chen H et al., who tested SARS-CoV-2 on throat swabs of eight newborns and samples from the breast milk of three mothers, no positive outcomes were reported [67]. In addition, an American study on 43 females found no SARS-CoV-2 infection among neonates on the first day of life [68]. Likewise, a systematic review of 41 pregnancies showed that most C-section deliveries had no symptoms of vertical transmission [60].

Post-delivery care

Maternal: Women must recover either in a theater or isolated delivery room instead of in a post-anesthesia care unit. After delivery, several patients show worsening indications and should be carefully monitored. If a woman has increasing requirements for oxygen or is in respiratory distress, she should be shifted to the ICU. Tubal sterilization, if required, should be delayed for 4-6 weeks once the patient has recovered completely from COVID-19. Additionally, postoperative visits should be performed electronically [69].

Fetal: A sample from neonatal nasopharyngeal suction before the first breath may be collected for coronavirus testing prior to prompt cord clamping. The decision regarding the temporary isolation of a mother from her infant should be made on a case-by-case basis after consulting clinicians, healthcare officials, and prevention and control specialists [70]. The decision should be made after assessing disease severity, signs and indications, and laboratory results for COVID-19. Women with suspected or confirmed infectivity should abstain from breastfeeding until they are completely recovered [70].

Breastfeeding or not: Regarding breastfeeding, among women infected with COVID-19 [30], the main risk is infants’ close contact with mothers, who are likely to shed infectious airborne droplets [71]. Chinese research reported no evidence of SARS-CoV-2 in the breastmilk of infected mothers [67]; thus, breastfeeding benefits seem to outweigh any potential risk of virus transmission [30]. However, for those desiring to breastfeed, safety measures should be adopted to reduce the virus risk to infants by observing proper hand hygiene before touching the baby. Mothers are also recommended to wear face masks while breastfeeding [30,71].

Psychological support: Pregnant women with COVID-19 are at enhanced risk for depression and anxiety; they may show varying levels of psychiatric indications that are harmful to maternal and fetal health [21]. Moreover, mother and baby separation can hinder early bonding and the establishment of lactation, both of which cause unavoidable further stress in women during the postpartum period [21]. Therefore, health professionals should pay close attention to patients’ mental health, including prompt evaluation of their sleep patterns and causes of depression and anxiety [21].

Weaning from mechanical ventilation

There is no consensus or guidelines for MV withdrawal in obstetric patients. However, patients should be removed from MV as soon as the cause of MV has resolved or if conditions have improved sufficiently for the patient to sustain spontaneous respiration without assistance [72,73]. The cuff leak test is a predictive method for post-extubation stridor due to laryngeal edema or a decrease in the cross-sectional area of the trachea. As obstetric patients present with general airway and laryngeal edema, it is necessary to perform this test before extubation. The test consists of deflating the cuff and observing the leakage in the volume-time curve. A difference in leak >20% or >110 ml concerning previously recorded by the ventilator is sufficient to tolerate extraction of the orotracheal tube, whereas a leak <20% indicates tracheal or laryngeal edema that will require immediate treatment and subsequent reassessment. To decrease laryngeal edema, corticosteroids are recommended before the cuff leak test and as treatment in the event of post-extubation stridor [74].

A failed cuff leak test does not mean that extubation should be delayed much longer. IV steroids should be started as soon as possible or four hours before intubation (usually methylprednisolone 20 mg every 12 h for three doses). The cuff leak test can be reassessed from the second or third dose, at which point withdrawal from MV can be considered again [73].

Special consideration

Cardiopulmonary Resuscitation (CPR)

CPR is an essential component of care that places saviors at an enhanced risk of contact [59]. Its administration involves executing practices that cause intubation, aerosol chest compression, and bag-mask ventilation. Therefore, health personnel should undertake all personal safety measures prior to CPR administration. Regarding the administration of CPR among pregnant women with COVID-19, no precise recommendations are available except for ensuring administrator safety [75]. It is highly suggested that all health personnel must perform simulation-based training to manage CPR after applying PPE. Health workers at the workplace must be limited to patients who require critical care. Rescuers must consider superseding manual chest compressions together with automatic devices if accessible. If intubation is delayed, a bag‑mask/supraglottic airway device along with a high-efficiency particulate air (HEPA) filter can be utilized [60,75]. Once spontaneous circulation is restored, the parturient should receive critical care in the ICU dedicated to COVID‑19. The indications and procedures for perimortem CS remain the same [59].

SARS-CoV-2 Vaccine During Pregnancy

The Joint Committee on Vaccination and Immunization approved vaccination for pregnant women based on their age and clinical risk cohort, according to non-pregnant women. No biologically possible mechanism is available through which any vaccine can lead to harm during preconception, gestation, or breastfeeding [76].

From June 2021, more than 120,000 pregnant women in America received the COVID-19 vaccine without any adverse effects [76]. The outcome of the Moderna or Pfizer-BioNTech vaccines has been reported in 3958 women during pregnancy or the periconception period [77]. At the time of reporting, 827 pregnancies were completed; among them, 712 were live births (out of 700 females vaccinated during the first trimester). The unfavorable outcomes (preterm delivery, stillbirth, small GA, and spontaneous miscarriage) were similar to those of the background population. In addition, neonatal mortality was reported, and in infants with reported inborn deformities, none of their mothers had received a vaccine during the periconception period or in the first trimester [76].

## Conclusions

COVID-19 is a leading public health dilemma that requires critical care, especially for pregnant women who are more vulnerable to COVID-19 complications because of physiological and immunological changes during gestation. Practical methods that can help in timely diagnosis, as well as treatment, can be used to protect pregnant females and their infants from severe COVID-19. Pregnancy management is a keystone to overcoming the epidemic, and delivery is an important trigger. Maternal home and prenatal care management, delivery timing, mode selection, delivery management, and later puerperal protection are essential for obtaining a healthy baby. Fortunately, most pregnant females with COVID-19 show good recovery, and full-term vaginal birth can be expected.
